# Comparison of Globus Pallidus Interna and Subthalamic Nucleus in Deep Brain Stimulation for Parkinson Disease: An Institutional Experience and Review

**DOI:** 10.1155/2017/3410820

**Published:** 2017-06-19

**Authors:** Shazia Mirza, Umar Yazdani, Richard Dewey III, Neepa Patel, Richard B. Dewey, Svjetlana Miocinovic, Shilpa Chitnis

**Affiliations:** ^1^Department of Neurology and Neurotherapeutics, UT Southwestern, Dallas, TX, USA; ^2^Department of Neurology and Neurotherapeutics, Section of Movement Disorders, UT Southwestern, Dallas, TX, USA; ^3^UT Southwestern Medical School, Dallas, TX, USA; ^4^Henry Ford Hospital, Detroit, MI, USA; ^5^Emory University, Atlanta, GA, USA

## Abstract

Deep Brain Stimulation (DBS) has revolutionized the lives of patients of Parkinson disease, offering therapeutic options to those not benefiting entirely from medications alone. With its proven track record of outperforming the best medical management, the goal is to unlock the full potential of this therapy. Currently, the Globus Pallidus Interna (GPi) and Subthalamic Nucleus (STN) are both viable targets for DBS, and the choice of site should focus on the constellation of symptoms, both motor and nonmotor, which are key determinants to quality of life. Our article sheds light on the specific advantages and drawbacks of the two sites, highlighting the need for matching the inherent properties of a target with specific desired effects in patients. UT Southwestern Medical Center has a robust and constantly evolving DBS program and the narrative from our center provides invaluable insight into the practical realities of DBS. The ultimate decision in selecting a DBS target is complex, ideally made by a multidisciplinary team, tailored towards each patient's profile and their expectations, by drawing upon scientific evidence coupled with experience. Ongoing research is expanding our knowledge base, which should be dynamically incorporated into an institute's DBS paradigm to ensure that patients receive the optimal therapy.

## 1. Historical Perspective

Therapeutic targets for ameliorating the disabling symptoms of Parkinson disease, namely, tremor, were discovered early in the 20th century by neurosurgical observations, leading to an era of ablative procedures targeting the basal ganglia, refined further by advancements such as stereotactic surgery. The advent of levodopa therapy in the 1960s resulted in a decline in surgical management; however, the eventual emergence of side effects and suboptimal control of medication related phenomena such as dyskinesias and motor fluctuations have renewed the interest in surgical interventions [1]. While the use of electrical stimulation of the nervous system for pain control and seizures has been in play from the early part of the 20th century, the role of DBS in Parkinson disease can be traced to the outpatient stimulation of the thalamus and globus pallidus for motor disorders in 1972 by Bechtereva, to the first implanted stimulator for tremor in a Multiple Sclerosis patient in 1980 by Brice and McLellan, and last but not least to the first reported case of DBS in Parkinson disease at the ventral intermediate nucleus of the thalamus in 1987, by Dr. Benabid's group in France [2, 3]. This was followed by a rapid exploration into the potential of DBS, resulting in a field continuously evolving and improving in its technique, technology, knowledge, and mandate [4]. The evolution of therapies for Parkinson disease involves an incredible journey of collaboration between clinical and basic sciences. A fortuitous observation linking Parkinsonian symptoms in young drug users resulted in the identification of MPTP [5], with the subsequent development of animal models [6] which allowed robust experimentation and proof of therapeutic targets for Parkinson disease [7–9].

## 2. Introduction and Evolution of the Program

The Deep Brain Stimulation program at UT Southwestern Medical Center in Dallas began in 1998 following FDA approval of DBS (in 1997 for essential tremor and 2002 for Parkinson disease), with steady growth in the number of procedures performed, up to 45 per year in 2016. The rapid evolution of the program mirrors the data available through various large studies, on the benefits of DBS versus medical therapy alone. Two landmark clinical trials [10, 11] demonstrated the efficacy of DBS over best medical management in improving motor functions, on-time without dyskinesias, and quality of life at 6 months, which have to be weighed against an increased risk of serious adverse events. While DBS does cause a significant improvement in motor scores as compared to medical management, this does not always translate into an improved quality of life, since complications associated with DBS surgery such as seizures and negative effects of DBS on cognition and mood may not allow the motor gains to be perceived and may in fact decrease quality of life. At UTSW, as part of a continuous quality improvement process [12], the DBS program collects data on both motor improvement and quality of life for analysis of patient outcomes, which will allow us as an institution to track our performance and ensure the best possible results.

Medical decision-making represents the art and science of weighing evidence based information with practice preferences, both comfort and experience, and target selection for DBS in Parkinson disease patients is no different. The availability of several studies and analyses on this topic, including some randomized controlled and blinded trials which allow a higher level of confidence in their results, provides objective and up-to-date information, which equips physicians to exercise greater scientific rigor into their decision-making process and importantly enables patients to consent with relevant prognostic data.

## 3. Target Sites

The main targets for DBS in Parkinson disease are GPi (Globus Pallidus Interna) and STN (Subthalamic Nucleus), with the balance between the two tilting back and forth in light of new evidence. This decades-long duel has its origins in the pallidal preference for ablative procedures which was swiftly replaced by an overwhelming preference for STN when several prominent studies backed its superiority [13, 14]. The first study with a side by side comparison of the two sites in 2001 showed significant motor benefits of DBS therapy at either site and led to its FDA approval for Parkinson disease. While this study was not designed to compare the two sites in theory, the authors set the precedent for STN preference [15]. This penchant towards STN in DBS has been reexamined in several rigorous trials [16–18] which did not uphold its dominance, allowing a “rematch” as aptly termed [19, 20]. These reports have culminated in a consensus in the field to move away from a ‘one size fits all' use of STN for the disease, to a target choice which is tailor-made to a patient's specific symptoms and profile.

## 4. Site Selection

At UTSW, the target for DBS surgeries for patients of Parkinson disease is selected during the neuromodulation committee meeting, a multidisciplinary board comprising neurologists (movement disorders specialists), neurosurgeons, a dedicated DBS coordinator, speech language pathologists, physical therapists, and neuropsychologists. This process requires a comprehensive list of preselection tests, scales, and procedures in addition to motor scoring such as preoperative neuropsychological testing, brain MRI, physical therapy assessment, speech, and swallow assessment, to determine eligibility and site and maintain a baseline record of parameters to compare outcomes [12]. In terms of symptom alleviation, patients are selected to undergo DBS based on their response to levodopa. A levodopa challenge test, with an improvement of 30% on the UPDRS III (Unified Parkinson Disease Rating Scale, motor score), is accepted as the best predictive factor for successful DBS outcomes [21]. At UTSW, this motor scale is performed and videotaped both off medications and after an effective dose of levodopa. It is important for patients to be counseled on the expected results of DBS on each of their symptoms (both positive and negative) in order to align patient expectations with outcomes. It is at this monthly meeting that patients' history and test results are reviewed to ensure suitability for the DBS procedure, and if so, what the optimal target would be, based on the symptom profile and operative constraints if any. This inclusive, multispecialty, on-the-spot input is invaluable for a comprehensive assessment, with a thorough evaluation of the risk-benefit ratio for each unique case. This meeting incorporates relevant and recent studies in its decision-making to ensure scientific due diligence, reflected by the due process followed at UTSW to evaluate patients on a case by case basis, moving away from the global trend of STN as the site for DBS. We have performed 30 surgeries with GPi as the target and 28 with STN target through our DBS program, with an overlap occurring due to switching or addition of the other target site for enhanced symptom control. Over the last 2 years, these numbers are 22 with GPi as the target and 18 with the STN target.

## 5. Comparison of the Target Sites

A comparison of the two targets encompasses various parameters, such as therapeutic benefits, mechanisms of action, and adverse effects.

### 5.1. Anatomy

The anatomical differences between the two sites, namely, their size and location, are the basis for some of the observed differences. The GPi is roughly 3 times the size of STN and thus requires a higher charge density. Studies show that the DBS stimulation settings in patients with pallidal stimulation are significantly higher in amplitude and pulse width as compared to STN stimulation (with no difference in frequency) which results in more frequent battery changes and higher opportunity for surgical complications [16, 22, 23]. However, the disadvantages of stimulation applied to a compact target such as the STN include a spread of current to neighboring circuits, resulting in increased number of stimulation related adverse effects. The smaller size of the STN makes it harder to place the leads exactly in the designated sensorimotor areas, possibly overlapping with the limbic or associative areas of the STN and could be responsible for the various cognitive and psychiatric side effects by activation of nonmotor circuitry [20].

### 5.2. Symptoms

#### 5.2.1. Motor Symptoms and On-Off Period

Motor control is the primary treatment goal of Parkinson disease management and both GPi and STN-DBS equally improve motor function [24, 25]. The UPDRS III is used universally as a scale to measure the motor improvement after DBS and is assessed in varying combinations, with medication off and on and stimulation off and on. In a randomized trial conducted to compare DBS at the 2 sites at Veterans' Affairs and university hospitals (the Veterans Affairs Cooperative Studies Program), the motor improvements with medications off and stimulation on showed no statistical difference between the effects of DBS at either target at time periods extending out to 24 months [16] and 36 months [17]. Both time periods showed a slightly higher improvement with the pallidal target and a slight worsening with STN-DBS when both medications and stimulation were on. However, this effect was exaggerated (pallidal improvement and STN deterioration) when both medications and stimulation were off. Different studies show conflicting results: a randomized trial with a 1-year follow-up period (NSTAPS) showed a greater change in motor scores in the medication off phase with STN-DBS versus GPi [23], with the 3-year follow-up showing the same result [22], but no differences between the 2 sites in the medication on group. The major advantage with DBS is the amount of time spent in the “on” versus “off” period, a significant disability faced by patients managed medically. While most studies confirm this advantage and peg it at 4–6 hours of time saved from the “off” phase, with no difference between the STN or GPi sites, this is a measure usually tracked by the patients themselves through a diary and/or part IV of the UPDRS which does not always allow a rigorous analysis [20].

At UTSW, the DBS coordinator utilizes several scales to assess the motor performance both prior to the procedure and at follow-up visits. This includes parts 1–4 of the Unified Parkinson Disease Rating Scale, with part 3 performed on and off medications in the following combinations: stimulation off, medications off; stimulation on, medications off; and stimulation on, medications on. Specific tests are used for DBS performed for nonparkinsonian conditions such as essential tremor or dystonias.

#### 5.2.2. Tremor

With respect to individual symptoms, resting tremor responds successfully to both GPi and STN-DBS [20, 26]. It is postulated that tremor may be more effectively controlled with STN-DBS in part due to the size of the nucleus allowing a more complete stimulation-coverage of the area, which may be insufficient for the larger GPi [19]. The exact location of the leads within these nuclei is being studied for optimal tremor control. While the resting tremor is often suppressed, a coexisting essential tremor may progressively worsen with time, which could be addressed by using the posterior subthalamic area (PSA) as a target site [27] or the ventralis intermedius (VIM) nucleus, often used as an additional site for suppression.

At UTSW, an interesting case report of a patient who did not experience optimal tremor control with bilateral STN-DBS was presented at the ANA (American Neurological Association) in 2015 and is possibly the first case of unilateral GPi lead rescue for tremor due to STN failure and stimulation related side effects [28]. The patient was a 59-year-old right handed man with a diagnosis of Parkinson disease made at UTSW 6 years ago, who presented for DBS evaluation with severe right sided tremors (the initial symptoms) and milder left sided tremors affecting both upper and lower extremities along with other typical Parkinsonian symptoms such as rigidity and bradykinesia (predominantly right sided), hypophonia, stuttering speech, and slow gait. Due to disabling tremors refractory to optimal medical management, the patient opted for bilateral STN-DBS within 2 years and initially experienced nearly complete resolution of his right sided tremors. However, he could not tolerate the long-term side effects of stimulation such as tingling, numbness, and incoordination along with an eventual loss of left sided tremor control; in addition, the patient stopped taking his medications due to meager benefit and adverse effects. Neuroimaging demonstrated a misplaced lead on the right side which could be responsible for the stimulation related adverse effects (SAEs). The patient, limited by his medication intolerance and symptom resistance, consented to undergo another DBS procedure, a right sided unilateral GPi rescue lead with the expectation of better tremor control with the alternative target. Postoperative programming resulting in optimal tremor control especially on the left could be achieved with dual right sided (both STN and GPi) along with left-STN stimulation. While there are a few recent case reports of GPi-DBS serving as a “rescue” lead for symptoms such as dystonia, behavioral features, and dyskinesias in patients with STN-DBS [29, 30], this particular case serves as an example for the use of GPi rescue leads for a STN-DBS refractory tremor. The addition of rescue GPi leads reflects possible mechanistic differences, such as complementary activation of the GPi, aside from its indirect stimulation via STN-DBS. The supplementary GPi lead could allow the activation of additional motor pathways which may not be accessed via the STN, without concurrent stimulation of limbic and associative fibers, thus eliminating unnecessary side effects, along with an element of its inherent efficacy for the alleviation of a symptom such as dystonia [28, 30].

#### 5.2.3. Rigidity and Bradykinesia

Rigidity and Bradykinesia are motor symptoms that respond well to DBS at both targets. A study (COMPARE trial) showed greater improvement in rigidity with a unilateral STN-DBS lead versus GPi lead at a 7-month follow-up time point [26] but no such significant advantage between the two sites could be found in bilateral DBS studies at 6 months [17] or 12 months [18]. While most studies do not show a difference between the target sites for improvement in bradykinesia [17, 26], some studies have shown an advantage of STN stimulation [18, 31].

#### 5.2.4. Dyskinesia

Dyskinesia suppression is achieved at either target through fundamentally different mechanisms, direct stimulation effects of GPi-DBS and medication reduction in STN-DBS. This difference is responsible for the dyskinesia suppression in STN-DBS in the absence of active stimulation contrasted to GPi-DBS, which requires active stimulation for dyskinesia control at 3 months. However, dyskinesia control in GPi-DBS can be seen at 12 months even with stimulation off and can be attributed to long-term effects on dopaminergic pathways [18]. While a study reported a difference between bilateral GPi versus STN-DBS (89% versus 62% improvement in dyskinesia, resp.), it was not significant [18], but it went on to set the precedence of accepting GPi as superior in dyskinesia reduction [19]. STN-DBS has been associated with an exacerbation of dyskinesias [25] and “brittle dyskinesias” unamenable to control by optimizing programming and medication, requiring rescue surgery with GPi [32]. The superior suppression of dyskinesias, independent from medication, allowing flexibility in dose adjustment (to prevent dose-reduction side effects) and the absence of extraneous dyskinesias put GPi in the lead for patients with predominantly dyskinetic symptoms.

While motor manifestations of Parkinson disease are often well managed medically and with DBS, there is a shift in the patient experiences towards other often disabling abnormalities of gait, posture, speech, cognition, mood, and autonomic disturbances, all of which are important determinants of quality of life.

### 5.3. Cognition

A decline in cognition significantly affects quality of life and is seen both in the natural progression of the disease and after DBS. The effects of DBS on cognition, mood, and behavior are extensively studied, with most studies revealing lower abnormalities after GPi-DBS as compared to STN-DBS, largely responsible for the “rematch,” a shifting away from the STN only approach.

The issues concerning cognition and DBS are multifold. Cognitive risk factors in patients do not serve as blanket exclusion criteria for surgery; rather they influence patient counseling for postoperative expectation setting and possibly target selection. Patients with preexisting dementias are usually disqualified from DBS surgery due to risk of worsened cognitive outcomes [33, 34]. However, in patients with mild cognitive changes, the decision to undergo DBS is a risk-benefit analysis between improved motor symptoms and likely cognitive worsening, which may in turn impact the overall ability to function and quality of life. Issues of competency to consent for this invasive procedure as well as the ability to participate during the surgery itself and keep up with the extensive follow-up testing are brought to the forefront in patients with diminished cognitive reserve.

At the 24-month follow-up of the Veterans Affairs Cooperative Studies Program, to compare bilateral DBS at both targets, results showed a slight decrease in neurocognitive function in both groups with a significantly greater decline in processing speed in the STN group, especially in the visuospatial domain [16]. At 36 months, this effect was maintained, albeit the Parkinson Disease Questionnaire-39 (PDQ-39) cognition subscale scores did not reach a significant difference between GPi and STN. However, testing such as the Mattis Dementia Scale and measures of verbal fluency (the Hopkins Verbal Learning Test) did show a significant difference between the two sites (favoring GPi) [17]. A long-term (10-year follow-up) study of STN-DBS demonstrated a 46% prevalence of dementia in patients, with no relationship to mortality, occurring about five and a half years after the surgery. It must be kept in mind that dementia is a known nonmotor complication of Parkinson disease with an overall prevalence of about 40% and this reaches as high as 83% in patients who have the disease for over 20 years [35]. The presence of dementia is heavily linked to the age of patients, thus making age of onset of Parkinson disease a compounding factor while comparing dementia prevalence after certain duration of disease. This could explain why the latter long-term study (where patients had early-onset disease) did not show as high as previously observed dementia due to skewing of the data [36]. Another randomized trial that compared the two sites with unilateral stimulation, with a specific emphasis on mood and cognition (COMPARE), demonstrated no changes between the two sites in semantic fluency but a greater decline in letter fluency in the STN group, which did not reach their predetermined level of significance [26]. This study also demonstrated that the decline in letter fluency in the STN group was irrespective of stimulation setting (location) as compared to GPi. A meta-analysis of the effects of DBS on cognition showed small decreases in overall cognition as well as in domains such as memory, attention, executive function, psychomotor speed following STN-DBS, and moderate decreases in verbal fluency both semantic and phonemic. GPi-DBS on the other hand showed only a small deficit in attention and verbal fluency [37]. This superior effect of GPi on cognition has to be balanced by the conflicting data from the 24- and 36-month follow-up of a trial (NSTAPS) which shows no statistical difference between the 2 sites on a composite score encompassing adverse effects of behavior, cognition, and mood [22, 23].

A randomized trial using bilateral STN stimulation with a constant current device had an interesting and seminal finding that the decline in verbal fluency noticed after STN-DBS occurred in both the activated (stimulation turned on) and inactive leads (implanted but never activated) allowing one to infer that these effects are likely due to surgery itself and possibly the surgical trajectory rather than specifically STN-DBS [38].

Relying solely on cognitive screens is not always adequately sensitive to detect the often subtle postoperative cognitive changes, which require an in-depth neuropsychological assessment consisting of a wide array of tests spanning all domains [39]. UTSW has taken the approach of gathering extensive data through a battery of tests administered by a qualified neuropsychologist prior to surgery. The preoperative tests include cognitive tests, tests of executive function, tests for attention and processing speed, language testing, memory test, visuospatial tests, and testing mood and behavior. Scores from each test are converted to a global score as well as individual domain scores, which along with qualitative analysis are used to prepare a graph enabling an easy visual interpretation of data at the monthly committee meeting. Postoperatively, cognitive function is tracked through the Montreal Cognitive Assessment (MoCA) and this along with surgical complications, motor, and quality of life data are discussed at the outcomes review meeting. This ensures that patients with suboptimal outcomes are immediately identified and corrective action, if any, can be undertaken [12].

However, despite preoperative cognitive screening, several patients go on to develop cognitive dysfunction including dementia, often within 6 months of surgery [34, 39, 40]. While dementia is part of the natural progression of the disease, surgery likely plays a precipitating role. Cases have been reported of immediate postoperative cognitive decline following STN-DBS, often associated with suboptimally placed leads [41, 42]. At UTSW, we have identified two such patients who presented with disabling cognitive symptoms soon after DBS [43].

A 67-year-old male patient living with Parkinson disease for 7 years with normal preoperative cognition underwent bilateral STN-DBS. Apart from mild global atrophy, minor word-finding difficulties, and slowed thinking, he could independently manage his finances and medications. After an uneventful surgery, he experienced relief of motor symptoms and had his medications reduced; however, cognitive changes were noticed by his family within a matter of days. He experienced prominent memory and executive function declines, including impulsiveness, disinhibition, poor judgment, and inappropriate behavior. His REM behavior disorder symptoms worsened and he was frequently angry. Neuropsychological testing after 2 months revealed frontal lobe dysfunction with significantly reduced problem solving, attention, memory, and phonemic fluency, compared to preoperative levels, unchanged by increasing his levodopa dose. Similar testing 14 months after surgery revealed global cerebral dysfunction, with major involvement of the frontal lobes, consistent with dementia. He was evaluated for undetermined spells for which EEG did not reveal abnormalities and was subsequently treated for orthostatic hypotension. The second patient was a 57-year-old man with Parkinson disease for 9 years, who underwent bilateral STN-DBS. He was preoperatively found to have mild global atrophy, past history of medication induced hallucinations, practically minor problems with memory, and mild frontal-subcortical cognitive deficits on testing, which were stable over a year. He experienced confusion after an uneventful surgery, which worsened after the battery placement. Despite motor benefits, he became inattentive and disoriented, with worsening anxiety. Neuropsychological testing 3 months after surgery revealed global decline, with prominent frontal-subcortical involvement, consistent with mild to moderate dementia. In both cases, a medical work-up was unrevealing and MRIs taken 1 month postoperatively did not show any signs of infection, hemorrhage, or infarct. A study of the lead trajectory revealed they all passed through the frontal lobe, lateral ventricle, and posterior-medial border of the STN, with the lead in Patient 1 travelling further caudal towards the midbrain-pontine junction after a year. The lead positions in the first patient were noted to be posterior-medial in the STN, rather than in the dorsal STN for optimal motor benefit, and at a much greater than intended depth. Turning the stimulation off for a few weeks did not halt the cognitive decline in the patients, which the authors suggest to be attributed to the lead positions, its trajectories, and the surgery itself hastening the process [43]. This highlights the necessity of stringent preoperative screening and counseling in patients with mild cognitive impairment undergoing DBS as both hallucinations albeit medication related and prior cognitive impairment (as seen in the second patient) are risk factors for this adverse outcome [40]. The quality improvement project has identified this as an area that would benefit from cycles of plan-do-study-act. With adequate postoperative MRI scans, it is possible to correlate clinical outcomes with lead locations and trajectories. This along with neurosurgical input would allow a plan to ensure more accurate lead placement and localization [12].

### 5.4. Mood and Behavior

Several patients experience stimulation related changes in mood and behavior such as depression, hallucinations, impulse control disorder, apathy, and dopamine dysregulation syndrome. These adverse effects may be related to preexisting psychiatric illnesses, stress, medication reductions, surgery-related factors, changes in social situations following surgery, and mismatched patients' expectations versus outcomes [34]. While these occur in patients of both STN and GPi-DBS, several studies have shown a preponderance of negative effects on these parameters with STN-DBS. A study of 24 patients with bilateral STN-DBS revealed that careful selection of patients was required to enjoy the motor benefits of DBS. A preponderance of anxiety and emotional hyperreactivity after surgery along with other undesirable behavioral side effects and maladjustment to the family or social environment resulted in unsatisfied patients, despite motor improvements [44].

A trial to compare the effects of unilateral DBS at the STN and GPi, specifically on mood and cognition (COMPARE trial), gathered data on the impact of DBS using Visual Analogue Mood Scales (VAMS) with several subscales. Seven months after surgery, there were an overall reduction of tiredness in both groups and improved scores in “feeling happy” and “less tense”; however, feelings of anger, confusion, and irritability increased. There was no significant difference between the two sites in these subscales of mood, except for an increased anger in the STN group [26]. Based on the position of the leads in this study, the most undesirable outcome (less happy and energetic, more confused, and sad) occurred in a ventral stimulation setting at both sites, which could be a possible explanation for patients who suffer severe mood changes due to incorrect lead placement.

The Beck Depression Inventory II, used as a measurement tool, showed an improvement in the GPi group contrasted to a decline in the STN group [17], with similar findings by a meta-analysis showing greater improvements in this scale in the GPi group [25]. However, the former study (Veterans Affairs Cooperative Studies Program) showed that the differences between the two sites disappear by 36 months [18]. While most studies concur on the greater negative outcomes of STN-DBS on mood and behavior, a randomized and blinded trial (NSTAPS), on the contrary, demonstrated no differences between the GPi and STN in a composite score, designed to measure various aspects of mood, cognition, and behavior, and the findings remained constant at the 12-month and 36-month follow-up [23, 24].

Changes such as delirium, hallucinations, anxiety, hypomania, and apathetic mood have been noted in the perioperative period, in patients who underwent STN-DBS with no similar changes noted in patients who underwent GPi stimulation [18]. While perioperative changes usually resolve, the existence of hallucinations prior to surgery must be evaluated while determining eligibility for the procedure. Drug-induced hallucinations are expected to improve due to dosage reductions made possible after STN-DBS, but those due to the disease itself are worsened and usually serve to exclude patients from the procedure. In a long-term (10 years) follow-up of STN-DBS patients, hallucinations were present in nearly 60% of the group. These hallucinations occurred approximately 4 years after the surgery, were associated with a higher mortality and the use of antipsychotics, and, importantly, showed no significant difference in the dopaminergic medication doses in those suffering from psychotic symptoms versus those who had no such symptoms [36].

There is a wide range of effects of DBS on impulse control disorders (ICD), ranging from complete resolution of preexisting disorders partial resolution and generation of new ICDs after DBS. A study following a group of patients with bilateral STN stimulation patterned the above, with 23% of the cohort having preexisting ICDs, of which 84% of them benefited from their resolution after DBS, and the rest had an appearance of new eating disorder symptoms. There is a strong association between dopaminergic medication dose and ICD occurrence, strengthened by the observation that reducing medications after STN-DBS correspondingly reduces the incidence of ICDs; however, this cannot explain the occurrence of new ICDs in patients with already reduced doses of medication following DBS. Of the cohort that did not have preexisting ICDs, 14% developed them transiently, a year after surgery, lasting for about 15 months, all of which disappeared at the 3-year follow-up. Compulsive eating disorders were the most frequently seen behavior after STN-DBS and could account for the weight gain seen after STN-DBS [34, 45]. The physiology of impulsiveness due to dopaminergic drugs is different from stimulation effects of STN-DBS and could be responsible for the new symptoms. Attempts to stabilize ICDs should be undertaken prior to the procedure, as DBS while providing relief in certain cases cannot be used as an indication for surgery [20].

A large concern with the neuropsychological changes seen after DBS is the increased possibility of suicide following STN-DBS. While suicide (attempts and completed) have been observed [34], a large study examining this did not show a statistically significant difference in the onset of suicidal ideation between the group treated with DBS as compared to the group treated with best medical therapy (1.9% for DBS versus 0.9% for BMT) [46]. This held true while comparing the suicidal ideation between patients randomized to STN or GPi-DBS at 6 months (1.5% versus 0.7%, resp.), albeit several of the proxy symptoms were worse in the STN group. The reasons for this are multifold: medical and neurological conditions and complications related to the disease and surgery, the often drastic reduction of dopaminergic medication (possibly accounting for the decreased risk in GPi-DBS), the preexistence of psychiatric comorbidities such as depression, and the change in impulsivity, all of which play a role in increasing the risk of suicidal ideation. This reiterates the need for careful preoperative neuropsychological assessment, continuous monitoring of depression, and careful observation for the emergence of impulsive behaviors and warning symptoms by family members.

A study analyzing the subjective or patient-perceived benefits following STN-DBS revealed negative outcomes in spite of almost universal motor benefits in patients who underwent bilateral STN-DBS. Older age and longer duration of disease were not associated with perceived negatives outcomes; rather, the main predictors were axial symptoms and apathy [47]. This study highlights apathy as the single most important contributing factor towards subjective negative outcomes after DBS, significantly higher in these patients at baseline (prior to DBS surgery) as well as at the 12-month follow-up, with similar findings for depression. The aggravation of apathy in patients receiving stimulation is independent of any changes in depression or cognition and could be related to stimulation adverse effects or medication reduction. While patients are usually counseled that their axial symptoms would not be controlled by DBS, they are often perceived to have worsened, which may be in part due to their progression after surgery or that patients focus on them upon resolution of other motor symptoms. This in turn affects the quality of life scores and their own perception of benefit after surgery, highlighting the absolute need for setting expectations with the patient and their caregivers clearly and repetitively.

Possible explanations for the greater deterioration of cognitive and psychological parameters in STN-DBS patients include the effects of anatomical size and medication reduction. Leads placed in the STN may spread the current into the associative and limbic regions of the nucleus as well as areas such as lateral hypothalamus, zona incerta, and medial forebrain bundle, all of which have extensive limbic connections. The role of dopaminergic medications which are dramatically reduced in STN-DBS patients but relatively maintained in GPi patients may play a part in the latter's cognitive advantage. This framework can be applied to understand impulse control disorders as an imbalance between excess of dopamine with stimulation of limbic circuits leading to their hyperactivity [21]. This necessitates a balance between reducing the dosage of medication and increasing the intensity of stimulation. A study of bilateral STN-DBS patients observed that it is possible that certain depressive disorders may be unmasked after the surgery itself. Patients with previously undiagnosed or unnoticed behavioral disorders experienced a decompensation after STN-DBS. This again highlights the need for extensive preoperative neuropsychological counseling, delving into relevant topics, such as present or remote addictive behaviors, personality disorders, and depressive disorders, and, importantly, an in-depth examination of the sociofamilial environment of the patient [44].

At UTSW, mood and behavior are evaluated with cognition prior to the procedure and at follow-up visits, as explained above. The battery of tests includes the Beck Depression Inventory II, the Questionnaire for Impulsive-Compulsive Control Disorders in Parkinson disease-Rating Scale (QUIP-RS), and the Quick Inventory of Depressive Symptoms (QUIDS). Mood and behavior changes are also captured in the quality of life data which is reviewed at follow-up meetings, albeit there is lack of formalized testing in these domains in the absence of patient complaints or adverse events. Armed with this knowledge, it is imperative that patients' caregivers play an active and ever-vigilant role in the assessment for subtle changes in the patient's mood, behavior, and affect, after the DBS procedure. At UTSW, we have incorporated realistic expectation setting in our preoperative consent forms [12] clearly explaining which symptoms are likely to improve, which ones are not expected to improve, and possible side effects. However, it is in our interest to ensure and recheck that the patients and their caregivers have a complete understanding of these points and are not overwhelmed by the extent of testing or holding on to unrealistic expectations.

### 5.5. Quality of Life

The aim of all therapy is to ultimately improve quality of life for patients; this especially holds true for interventions such as DBS which are invasive and expensive and potentially have serious adverse effects. While controlling the motor symptoms of Parkinson disease is the primary goal of medical and surgical therapy, nonmotor symptoms such as mood, cognition, sleep, autonomic dysfunction, speech, and swallowing deficits form a large part of the disease burden and are important determinants of quality of life. These symptoms are more often than not resistant to medical and DBS therapy and often negatively impact patients' perception of therapy, despite the control of the cardinal motor symptoms.

Parkinson Disease Questionnaire, PDQ-39, is almost universally used as a measure of quality of life across 8 domains or subscales: mobility, activities of daily living, emotional well-being, social support, stigma, cognition, communication, and bodily discomfort.

A trial comparing bilateral DBS at the 2 sites (NSTAPS) did not find a significant difference in the quality of life measured through the Parkinson disease quality of life questionnaire-PDQL, not only at the 12-month follow-up but also at the 36-month follow-up [22, 23]. The Veterans Affairs Cooperative Studies Program after 24 months found the quality of life as measured by the PDQ-39 improved in most domains in both groups, with no significant differences between them. Although there was a minor deterioration in communication in both groups and worsened social support after pallidal stimulation versus improved support after subthalamic stimulation, these were not statistically significant, with an overall positive impact on quality of life [16]. However, by 3 years, these quality of life gains were diminished, with scores returning to baseline in certain domains such as emotional well-being, social support, and cognition, with no differences between the 2 sites [17]. Activities of daily living followed a very similar trend, which did not show sustained gains at 3 years after DBS, despite motor improvements. This loss of benefit is important to note while counseling patients on DBS. A randomized trial studying the effects of unilateral DBS GPi versus STN on mood and cognition allowed an in-depth analysis on the impact across various subscales of quality of life, 6 months after surgery [48]. With similar improvements in motor and mood symptoms, patients who underwent GPi-DBS reported a significantly higher quality of life as compared to those who underwent STN-DBS, with both groups showing improvement in 6 subscales (mobility, activities of daily living, emotional well-being, stigma, cognition, and bodily discomfort) but not on social support or communication. The level of depression measured by the Beck Depression Inventory II was predictive of overall quality of life improvements as well as the performance on the subscales of emotional well-being and support. The decline in category fluency was also correlated with a decline in the communication scale in the STN-DBS group. The overall impact found by this study, albeit with unilateral DBS, is an improvement in the quality of life at both target sites, markedly higher in the GPi-DBS group, affecting the various domains differently.

Nursing home placement is another component, intimately linked to ability to carry out activities of daily living and quality of life. A study which followed the long-term performance of STN-DBS across a span of 10 years found 42% of their patients were admitted into a nursing home, and this had a correlation with higher age at the time of surgery [36]. Parkinson disease patients have a higher risk than the general population to be admitted into a nursing home, linked to their duration of disease, age, and dementia, but studies have also shown that nursing home placement is less in DBS treated patients as compared to medically managed patients alone (6% versus 15%) [49].

Recently, studies have reviewed the PDQ-39 as the standard questionnaire used to assess quality of life across the various disease specific domains. Despite almost universal usage including our institution, it has its drawbacks. It does not capture various side effects seen with STN-DSB, such as apathy, speech difficulties, and impulsive behaviors and underreports axial symptoms. The PDQ-39 was designed prior to this data being available and cannot comprehensively outline the extent or magnitude of benefit or impairment experienced by the patients. Since apathy plays a major role in the patients' perceptions of benefit, the quality of life scale ought to include apathy in its computation [47]. It is essential to have a scale that reflects all the known parameters which are affected by DBS and to that effect a group has worked to develop and validate a new deep brain stimulation impairment scale (DBS-IS). The scale consists of 22 questions for 6 subscales, with a high reliability and validity. The subscales include postural instability and gait difficulties, cognitive impairment, speaking problems, impulsivity summed score, and difficulties related to the DBS device. This DBS-IS is not designed to replace the PDQ-39, rather it is complementary to it and can assist in DBS candidate selection; for instance, high preoperative apathy or postural and gait imbalance scores may caution against the procedure.

At UTSW, the PDQ-39 is measured by the DBS coordinator both prior to surgery and during follow-up visits. We have not incorporated this new scale in our practice at UTSW and will probably wait for a refined version which addresses some of the drawbacks. These limitations include being designed exclusively with STN-DBS patients, possible missing other target specific symptoms, as well as being constructed with patient and care-giver experiences over a period of 1 year after surgery, which may not accurately represent or capture the long-term experiences with the DBS procedure [50]. It is important to note however that this group addresses a clinically relevant gap and the extensive preoperative testing and follow-up performed at our institute can incorporate these parameters.

### 5.6. Gait and Balance

Improvements in gait and balance mirror the effects of levodopa in the “on” period, which is increased by DBS. However, this gain is often lost due to progression of the disease, possibly hastened by surgery [40] and lesional effects of the procedure itself. It is vital to counsel patients on the ineffectiveness of DBS on medication unresponsive gait and balance issues. At UTSW, automated gait and balance assessments (in the medication “on” and “off” phase) using the APDM Mobility Lab (consisting of up to 6 wireless sensors on the patient, to measure the various kinetic parameters during predefined tasks such as walking and turning, which are analyzed using various plug-ins such as iTUG and iSWAY) provide objective measures of gait and sway [12]. This testing is carried out both pre- and postoperatively. A randomized trial at the veteran's affairs and university hospitals showed the superiority of GPi over STN-DBS for gait issues when both medications and stimulation were off, which lasted for an extended period (24 months) [16] with conflicting findings in another trial (NSTAPS) which showed STN-DBS superiority for gait in the off phase in a post hoc analysis [23]. Experimentation using DBS at the pedunculopontine nucleus (PPN) as an alternate management site for gait and balance instability is underway [51], yet to be performed at UTSW pending stronger safety and efficacy data.

### 5.7. Speech and Swallowing

Axial functions such as speech and swallowing are complex functions and have a direct relation to mortality (by aspiration) and need to be addressed for optimal quality of life. A review of the effects of DBS on swallowing highlights the fact that while most studies suggest an impairment of swallowing with STN-DBS, there was no clinically significant impairment or improvement measured, and studies notably did not compare STN to GPi or unilateral versus bilateral stimulation [52]. STN-DBS has been reported to help reduce the vocal tremor in patients with a trade-off of reduced volume, and DBS induced dysarthria, possibly due to the spread of stimulation to the corticospinal tract, ultimately reducing speech intelligibility [20, 53]. At UTSW, the effects of DBS on these functions are recognized and their comprehensive assessment is part of the preoperative work-up for patients. This involves a swallow evaluation and laryngeal video stroboscopy and performing the Consensus Auditory-Perceptual Evaluation of Voice (CAPE-V) and the Voice Handicap Index (VHI-10). This potential deterioration of speech and swallow functions after DBS is addressed while counseling patients for the procedure and specific treatments if possible are instituted before the surgery. Ideally we would make speech and swallow testing part of the postoperative work-up to track progress and enable early detection of deterioration if any, but since we are limited within the framework of insurance, this testing is performed postoperatively only in cases with reported adverse events.

### 5.8. Autonomic Symptoms

Patients with Parkinson disease often suffer from autonomic symptoms; their response to DBS remains variable and understudied and mainly in STN targets. While STN-DBS was thought to impact blood pressure and heart rate, studies have not demonstrated statistical changes. The positive effect of STN-DBS on constipation is noted but this is likely to be related to increased patient mobility. A direct effect of STN-DBS on bladder dysfunction has been theorized but is not clear [21].

### 5.9. Sleep

STN-DBS has demonstrated an improvement in the quality of sleep for patients, with an increase in total sleep time, time spent in REM, and slow wave sleep as measured by polysomnography and the PDSS: Parkinson disease subjective sleep scale. The improvement in scores was noticed when stimulation was off and can be attributed in part to the surgical lesioning of the STN nucleus itself. These effects were related to the extent of motor gains and the reduction of day time sleepiness due to reduced medications, thus improving night time sleep quality. The few studies tracking this data have not shown a difference between the two sites in sleep benefits [23]. Similarly, there is limited and conflicting evidence on the effects of STN-DBS on apathy and fatigue, from mild improvement to worsening, possibly related to the effects of decreased medication, with a possible emergence of fatigue as a long-term complication of STN-DBS [21, 54]. At UTSW, we have not incorporated the PDSS or polysomnography into the DBS program design for outcome tracking but are open to its utilization pending clinical need.

### 5.10. Pain

Patients of Parkinson disease experience different types of pain: musculoskeletal, dystonic, central, radicular, and somatic, exacerbated during the off period. The relief afforded by DBS to motor symptoms such as rigidity and dystonias is largely responsible for ameliorating the first 2 categories and possibly acting on central pain as well. A paradoxical effect of body discomfort is observed infrequently in patients particularly of STN-DBS as the reduced blood levels of levodopa result in a decreased pain threshold [21]. At UTSW, patients' pain and discomfort are tracked in the Parkinson Disease Questionnaire-39 (PDQ-39), a self-assessment of various quality of life parameters including bodily discomfort.

### 5.11. Mortality

It is worthwhile to investigate whether the improvement in quality of life and motor function in patients with Parkinson disease has an overall impact on mortality: is there a change in the natural progression of the disease with DBS?

Patients with Parkinson's disease have a higher mortality than the general population, with an odds ratio of 2.56 and a 5-fold higher chance of being placed in a care facility. So far, while drug therapies in some studies have shown a positive influence on the disease when started early on, they have not yet shown any change in mortality or the ability to prevent the onset of dementia or falls [55]. On the other hand, a trial exploring this question demonstrated that patients who underwent bilateral STN-DBS had significantly longer survival and were also significantly less likely to be admitted in a residential care facility (6%) as compared to matched patients (15%) who, while eligible for DBS, opted to be managed medically [49]. Another outcome observed in this study was a large cohort of patients in the medically managed group who died of respiratory causes as compared to the DBS group (20% versus 2%), likely related to aspiration due to swallowing impairments. This suggests that improved deglutition is a benefit of STN-DBS, with a favorable mortality advantage (albeit this study focuses on a STN target). Another long-term STN-DBS study (10-year follow-up) demonstrated that mortality had a 2-fold increase with an older age at the time of surgery (above 60 years) as well as a 9-fold increase in men. Surprisingly, the duration of disease or its severity or response to medications was not correlated with mortality. A higher age at surgery was also shown to be associated with increased nursing home placement [36].

A survival gain with DBS is usually not discussed with patients while considering them for DBS, information which should be incorporated into decision-making. At UTSW, while there is no fixed age cut-off for surgery, it is a factor discussed while weighing the risk to benefits of DBS in a particular patient. We have not formally tracked mortality data on patients who have undergone DBS at our center. So far two patients with DBS (one STN and one VIM (ventralis intermedius)) have passed away, but circumstances around their death involved multiple compounding factors and cannot be solely attributed to DBS. It is important for our quality improvement endeavors to have this data to allow outcomes tracking, and steps to ensure completeness of our database will be put in place soon.

Armed with the data showing that increased age at surgery is a risk factor for suboptimal outcomes and evidence that opting for DBS at an earlier stage of the disease rather than after exhausting all options is associated with superior motor and quality of life outcomes [56], a trend is emerging towards changing the age consideration for DBS surgery. At UTSW, the average age at surgery is 73 years for STN and 66 years for GPi. It also revises the treatment paradigm and presents the opportunity to begin consideration for DBS at an earlier time period. The ability to have a unified transition to surgical therapeutic options if required is a draw for several patients with Parkinson disease at UTSW, with in-house multidisciplinary teams available for seamless coordination of care.

### 5.12. Medication Reduction

There is a unanimous finding that medication (measured as levodopa equivalent dose (LED) or LEDD (levodopa equivalent daily dose)) is markedly reduced after STN-DBS as compared to a slight reduction after GPi-DBS. Studies have shown varying reductions such as 38% for STN-DSB versus 3% for GPi-DBS [18] or absolute dose reductions of 408 mg in the STN-DBS group versus 243 mg in the GPi-DBS group [16] at 2 years after surgery, which, despite slowly increasing by 36 months [17], remains significantly reduced as compared to baseline. While this is not the primary goal of surgery, this reduction in medication allows respite for patients suffering from disabling side effects which affect quality of life such as orthostatic hypotension, drug-induced dyskinesia, and fluctuations in “on” and “off” time. The reduction in medication should be managed cautiously, as a rapid reduction in certain medications such as dopamine agonists could lead to dopamine agonist withdrawal syndromes (DAWS).

The assumption that the reduction in medications is a pure positive effect must be examined, with evidence highlighting the complex interplay between symptoms, side effects, target stimulation, and medications. The possible increased suicidal ideation observed after STN-DBS has been linked to a reduction in dopaminergic medication [1]. Observations demonstrate the loss of prior positive effects of STN stimulation in the medication “on” phase especially for gait and balance, with worsened motor scores at extended time points (3 years after surgery) as compared to baseline [17]. This, coupled with the deterioration of motor scores in STN-DBS patients in the “off-off” (both medications and stimulation) phase, not seen in GPi-DBS patients which retain stable scores [57], bolsters the theory of dopaminergic medication advantage, and not merely disease progression as a possible explanation for these phenomena. This leads to various lines of thoughts such as the desirability of medication reduction in the absence of side effects, the nature of the relationship between medications and stimulation, whether STN stimulation interferes with dopaminergic stimulation, and whether there is an inherent disease modulating effect of GPi-DBS outside the role played by medications [58].

## 6. Unilateral Leads

Asymmetrical symptoms are a hallmark of Parkinson disease, and although uncommon, patients with predominantly unilateral symptoms have the option to undergo a single lead placement. A trial to compare the effects of unilateral DBS at the GPi and STN on mood and cognition (COMPARE) found no significant difference in motor and cognitive outcomes between the 2 sites, with differences in verbal fluency noted in STN-DBS when not in optimal settings [26]. A follow-up of these patients after 6 months reveals that more than half (52%) opted for implantation of a second lead for better management of their motor symptoms. Of the half that chose to remain with a single lead, two-thirds of them had GPi implantation. Thus, factors such as STN site lead and a lower asymmetric score were associated with a higher risk to convert to a bilateral implantation, in addition to worsened motor function (high UPDRS III scores), gait dysfunction, and dyskinesias. A possible explanation for this could be the different mechanisms of action of the two nuclei. Since GPi directly suppresses dyskinesias, it may serve to affect the contralateral side as well and continue to exert effects even without medication reductions, which makes unilateral GPi an attractive option for patients with severe one-sided dyskinesias. Bilateral STN leads are associated with greater medication reduction than unilateral leads; hence, adequate control of dyskinesias often requires both leads. Patients with predominantly unilateral symptoms could achieve motor control with a single lead, reducing the perioperative morbidity and side effects associated with bilateral implantation, and retain the ability to convert to bilateral leads when required [59].

## 7. Programming Paradigms at UTSW

Through a systematic process of testing each electrode, threshold settings are determined which elicit clinical benefit and adverse effects which are used in future programming. The stimulation settings are determined by varying voltage and pulse width along with contact points and occasionally frequency, all of which require a high level of skill to optimize thousands of combinations to attain symptom relief, with minimal DBS side effects. Programming offers the ability to manipulate advanced stimulation settings to achieve best possible outcomes even in cases with suboptimal lead placement. DBS programming and medication titration are delicately intertwined and require close monitoring until the patient experiences a stable and optimal state, a process that usually takes around 6 months. Following an optimization of settings, it is usually safe to expect that other Parkinson disease related effects cannot be managed by tweaking the DBS settings. It is also possible to anticipate DBS failures, early in the course of programming, in the case of low thresholds for adverse effects or unsustained benefits associated with a lead [1].

## 8. Surgical Complications and Adverse Events

Most studies and DBS programs track adverse events often with additional classification into mild, moderate, or severe to assist in analysis and comparative studies. The source of these events can be related to surgery, the device, stimulation, and medications or due to the progression of the disease itself and are not often distinguishable. It is observed that patients treated by medication alone have a higher frequency of adverse events overall; however, the patients with DBS experience a higher frequency of serious adverse events [10].

Surgical complications include infection (4%), intracranial hemorrhage (4.4%, leading to 1% rate of permanent neurological deficits), device related problems such as migration of leads (2.4%) and lead fracture (3%), and seizures (3.2%) and are considered major or serious events [21]. While the incidence of surgical complications may be considered equal between GPi and STN, it is theoretically possible that GPi, with a shorter battery life which may require more frequent battery changes, predisposes to higher infection rates. Adverse events were more common in the initial year after the surgery as compared to the following years, suggesting a favorable long-term safety profile aside from initial perioperative risks [60]. The Veterans Affairs Cooperative Studies Program showed no difference between the types (severity) or frequencies of adverse events at the two target sites, with most severe events resolving by the 24-month mark [16]. A study of bilateral STN-DBS with a constant current device demonstrated that while certain side effects such as fatigue and dysarthria were related to stimulation, others such as gait dysfunction and dyskinesias were present in the absence of the leads being activated, indicating a possible correlation to the surgery and/or tract itself [38]. Another surgical factor is the tract followed by the DBS lead during implantation; while it is inherently different for each target, it plays a role in the development of side effects independent of the effects of stimulation. A study showed a direct relation between the overlapping of electrode trajectories with the caudate nucleus in STN-DBS patients and the decline in cognition and memory [61]. Optimizing this trajectory and ensuring accurate lead placement are vital to achieve minimal side effects.

Certain side effects such as dysarthria were notably seen in the first 12 months after surgery, implying a relationship with DBS rather than progression of the disease. Other such symptoms linked to the DBS include dysphagia, excessive salivation, blepharospasms, and weight gain [36]. A randomized and blinded trial between the 2 sites noted the presence of perioperative complications in mood and cognition predominantly in the STN-DBS group such as delirium, hallucinations, and anxiety which resolved with time, but cognitive changes remained persistent [18]. The impact of DBS on various motor and nonmotor effects, such as impulse control disorders, depression, and worsening cognition, has been described in the relevant sections.

As part of the quality improvement initiative at UTSW, adverse events and complications, either minor or transient, are recorded to allow for an analysis of trends if any and devise improvements by the neuromodulation network meeting. This analysis is possibly the next quality improvement study (cycles of plan-do-study-act) which will be undertaken at UTSW by the movement disorders section in an effort to continually optimize our outcomes. There were a total of 9 surgical or device related complications in 28 patients studied, occurring in DBS performed at the STN as well as the VIM (ventralis intermedius) nucleus of the thalamus over the last 2 years [12].

## 9. DBS Failure

Suboptimal results from the DBS procedure are often labeled as DBS failures. Factors common to these “failures” include inadequate presurgical screening, improper patient selection, incorrectly placed leads, suboptimal programming, battery failure, and hardware related issues [1]. Patients are likely to switch their provider and DBS center in the hope of improved outcomes. Treatment options include optimizing the programming and medications to manage side effects or enhance benefits, as well as second surgeries, for correcting lead location in case of lead migration or incorrect placement or placing rescue leads at the other target site, which could be unilateral or bilateral.

A randomized trial comparing DBS at the 2 sites (NSTAPS) had 8 patients of GPi-DBS (from a total of 65) who had to undergo STN-DBS resurgery due to lack of benefits. Of these 8 patients, 5 had leads placed correctly. Similarly, 1 patient of STN-DBS (from a total of 63) had a unilateral GPi lead implanted, and one was reoperated to correctly position the leads [22]. A waning of prior positive response to GPi-DBS after a few months to years has been noted in several cases from the 1990s, which may be indicative of a surgical technique, patient selection, or postoperative management issue and often lead to surgical implantation of STN leads [62]. However, for the most part, studies showed long-term stability of GPi-DBS and STN-DBS [17]. The case report from UTSW on suboptimal tremor control with bilateral STN-DBS [27] discussed above is an example of DBS failure, due to improper lead placement.

## 10. Cost-Effectiveness

Since DBS is an expensive and long-term undertaking, it is important to discuss the cost-effectiveness of the procedure and accurately identify patients who will benefit from it given the risks and benefits. A study using the University HealthSystem Consortium (UHC) Clinical database found the average DBS procedure cost to be $39,152  ±  $5340 [63], with the median cost of implantation in 2013 being $34,052 at UHC-affiliated hospitals.

A study of the cost-effectiveness of DBS with medical therapy as compared to the best medical therapy (BMT) using various analytical models from the Medicare payer perspective, considering a 10-year horizon, found total costs of DBS to be $130,510 versus $91,026 for BMT, with a gain of 1.69 QALYs more than BMT, and a total of $23,404 per QALY, with greater benefit seen in younger age and longer follow-up [64]. A similar study in the UK pegged this at £20,678 per QALY gained [65]. The cost of the surgery is partially offset by the reduction in medication costs, for which STN-DBS with its drastic reduction shows a higher benefit. Expensive medication delivery routes such as continuous intestinal infusions of levodopa show a maximum cost benefit after surgery. In a randomized, single-blinded clinical trial to compare the differences between DBS with medical therapy versus medical management (ODT) in early stage Parkinson disease, an analysis of the cost savings showed that while drug costs increased 72% in the ODT group, they declined by 16% in the DBS + ODT group from baseline to 2 years. This difference resulted in a saving of $7150 per patient with DBS over the 2 years, which when extrapolated for the long-term (10 years) resulted in savings of $64,590 [66]. With a longer battery life likely related to programming characteristics STN-DBS shows a possible economic advantage with fewer surgical procedures required for battery changes.

We at UTSW have not undertaken a cost-benefit analysis, as costs depend on and vary with differing patients' insurances which skew the relevant data; however, it is a topic that will be researched to understand the financial implication of DBS, for both the patient and the institute.

## 11. Unanswered Questions

While DBS is widely used in the management of Parkinson disease, there are several questions that remain open, and the quest to solve them will hopefully strengthen the success of DBS in improving the quality of life in patients.

Questions regarding unilateral versus simultaneous bilateral lead implantation and the utility of the staged operations require further studies to determine benefit one way or another. The safety of unilateral implantation with concomitant decreased operative adverse effects especially in the elderly has to be weighed against the possibility of a second procedure for inadequate benefit and is usually considered in patients with extreme asymmetry of symptoms.

Issues faced by patients such as gait-freezing and other axial symptoms not addressed by DBS require studies to provide potential solutions. Research into stimulating multiple leads at different locations to address specific DBS and Parkinson's related symptoms is underway.

With increasing evidence for DBS at an earlier stage in the disease for superior outcomes [56], there is a need to define optimal age range and disease duration to experience the maximum benefit from this procedure. A recent prospective, randomized, single-blind clinical trial in early Parkinson disease (on medications for 1–4 years only) compared the motor and quality of life outcomes of the groups on optimal drug therapy (ODT) versus DBS with ODT. The study revealed that patients managed medically are 2–5 times more likely to experience clinically important worsening than the patients treated with DBS + ODT [67]. These results increase the options available to patients in the early stages of Parkinson disease and highlight the need to reexamine the therapeutic guidelines in place today.

Cases of DBS failures corrected by dual stimulation of alternate targets, the “rescue leads” as discussed above [28–30], point to the need for larger trials on the safety and efficacy of using dual stimulation as a therapeutic option in patients of single target DBS with inadequate symptom control due to SAEs or disease progression. This dual stimulation of both the target sites could also be considered as a potential DBS treatment strategy from the get-go, based on the symptom and patient profile, and needs further exploration.

While a majority of studies have an end point of 24 or 36 months, there are scarce long-term studies that have tracked patient outcomes over a span of 10 years [36]. With a possible trend emerging to perform DBS early in the course of the disease, it is imperative to have multiple studies addressing the long-term sequelae of DBS, both positive and negative, and to assess the impact on disease progression. This in turn may refine the patient and target selection and provide information for enhanced decision-making, for both patients and providers while analyzing the risk-benefit ratio.

## 12. Future Directions

DBS has become standard of care for treating patients living with Parkinson disease as well as a wide range of other movement disorders. The aim of research in the field is to have a clear understanding of the mechanisms of action of DBS and the physiological and anatomical basis of various side effects seen, so as to design a DBS process which will allow patients to achieve maximum control of all their symptoms (including ones traditionally resistant to DBS) with minimal side effects to enhance the quality of life. Research in this field has resulted in several potentially and therapeutically beneficial outcomes such as expanded scope, new targets, enhanced surgical techniques allowing precise target localization and trajectory such as frameless, and nonmicroelectrode recording techniques, advances in imaging such as functional and microstructural imaging, refined hardware design such as constant current devices, optimized programming sequences, and other cutting-edge techniques.

Continued research to elucidate the mechanisms of action of DBS at different targets and charting the anatomical zones and fiber bundles to explain corresponding side effects observed are important aspects of DBS research to understand and avoid the neuropsychiatric effects that accompany this procedure. Refining technical aspects such as contact selection may provide a solution to avoid these unintended effects [68]. Advances in stereotactic localization and imaging have made it possible to obtain accurately placed electrodes without patient participation, precluding the need for “awake” surgeries and allowing patients who are not ideal candidates for such “awake” procedures to benefit from DBS performed under general anesthesia, termed “asleep DBS.” “Asleep DBS” has been studied and shown to be both safe and effective, affording the patient significant motor benefit at 6 months after surgery [69], with no significant differences in the complications, hospital stay, or 30-day readmission rates as compared to the traditional awake surgery [70]. The availability of data from a meta-analysis on this question, showing similar efficacy and lower complications overall between awake and asleep DBS [71], makes this a viable option to include in the monthly neuromodulation committee meetings.

Research into new targets is underway and can potentially address the gaps in therapy with DBS at the STN and GPi. Studies on the pedunculopontine nucleus (PPN), chosen for its anatomic connections with the basal ganglia and its functional role in motor modulation and locomotion, demonstrate improvements in gait and balance including gait-freezing, the results of which are variable and need to be confirmed through rigorous trials. The PPN poses surgical access complications and has zones with varying functions; hence, studies need to address technique as well as costimulation of other targets [72]. Attempts to address gait and balance impairments using dual STN and Substantia nigra pars reticulata stimulation show promising effects on gait-freezing, with no additional effects on balance, mood, or cognition, and are being investigated in clinical trials [73]. Stimulation of the intralaminar thalamic complex comprising the parafascicular and centromedian nucleus is being evaluated for possible therapeutic benefit owing to its location in the basal ganglia circuit and projections to the striatum. DBS at this location modulates thalamocortical circuits with a positive effect on tremor [74].

Studies incorporating stimulation of novel targets in addition to the standard STN or GPi provide a theoretical means of addressing adverse effects or levodopa unresponsive symptoms. Experiments on the reward circuitry could address issues such as apathy, depression, and possibly other ICDs [75]. Through discovery of neural networks and rigorous trials, a possibility of multiple electrodes at different targets to address the various motor and nonmotor symptoms may be a distinct possibility, which along with real-time closed-loop programming may provide a dynamic control of a majority of symptoms, optimizing the patient's quality of life [20].

## 13. Conclusion

Deep Brain Stimulation has revolutionized the lives of patients living with Parkinson disease. The very decision to undergo the procedure and the choice of the target site are in essence a series of risk-benefit analyses. This entails balancing the probability of improved quality of life, relief of tremor, rigidity and other motor symptoms, reduced medications and their side effects with the possibility of potentially fatal or disabling surgical complications and other procedure related adverse effects, such as worsened cognition or other negative psychiatric outcomes, and changing the extent of impact of declined verbal fluency. Computing the best outcomes in these complex scenarios should be done on a case by case basis, using each patient's symptom, disease, medical, risk, and demographic profile along with patient expectations to reach the optimal management plan. This tailored approach to selecting the target site for patients undergoing DBS is the result of years of data from various studies, trials, and case reports, which allows a DBS team, including the neuromodulation network at UTSW to anticipate potential adverse outcomes and plan ahead to circumvent them, for the best possible results. If a patient has a history of hallucinations or has diminished cognitive reserve, GPi-DBS may provide motor benefit without the possibility of cognitive decline, if patients suffer significant medication related dyskinesias; STN-DBS can offer relief. In a similar vein, studies have also helped in planning the lead location and trajectory, with leads in the ventral zone of the STN, associated with a greater incidence of adverse effects. This planning and refining of patient and target selection, surgical trajectory and lead localization by imaging or microelectrode recording, DBS programming, and other hardware parameters have resulted in optimizing patient outcomes and unlocking the full potential that DBS has to offer. Research endeavors aim to continually enhance the DBS process by addressing the unmet needs and unanswered questions, through expansion of our knowledge based on the process mechanisms itself, technological improvements, trials to test or observe the safety and efficacy of various therapeutic options in DBS, and other novel ideas.

An indicator of the commitment of a DBS program towards its patients is the standard it holds itself up to and the accountability it has towards all its stakeholders. At UTSW, the quality improvement effort spearheaded by the movement disorders group in the department of neurology fulfills these obligations by subjecting various aspects of the program to a rigorous examination, cycles of plan-do-study-act, with the intention of ensuring these high standards. This endeavor has resulted in the design of the current processes, namely, the neuromodulation network and the formal structure in place for preoperative assessment and follow-up. An important element of this program is capturing data to allow tracking of outcomes and feedback [12]. The ultimate goal of these data-driven initiatives is to ensure that the DBS program dynamically processes and incorporates information to optimize outcomes in an agile environment. The commitment of the institutional leadership itself towards the success of the DBS program should be highlighted, as their financial support for the newly implemented processes is vital. The combined dedication of all the stakeholders to ensure the best possible patient outcomes is a testament of resolving of UTSW towards improving the lives of those living with Parkinson disease by developing safer and better DBS paradigms.
